# Clinical and health economic impact of isavuconazole for treatment of invasive aspergillosis and mucormycosis: a retrospective, matched multicentre cohort study in Germany

**DOI:** 10.1007/s15010-025-02674-x

**Published:** 2025-10-31

**Authors:** Marie Engelhard, Sebastian M. Wingen-Heimann, Beate Grüner, Maria J.G.T. Vehreschild, Johanna Kessel, Sabine Ehrlich, Karsten Spiekermann, Enrico Schalk, Ben-Niklas Baermann, J. Janne Vehreschild, Sina M. Pütz

**Affiliations:** 1 Faculty of Medicine, Department I of Internal Medicine, University of Cologne, University Hospital Cologne, Cologne, Germany; 2 Institute of Translational Research, Cologne Excellence Cluster On Cellular Stress Responses in Aging-Associated Diseases (CECAD), Faculty of Medicine, University Hospital Cologne, University of Cologne, Cologne, Germany; 3 Department I of Internal Medicine, Center for Integrated Oncology Aachen Bonn Cologne Düsseldorf (CIO ABCD) and Excellence Center for Medical Mycology (ECMM), Faculty of Medicine, University Hospital Cologne, University of Cologne, Cologne, Germany; 4 German Centre for Infection Research (DZIF), Partner Site Bonn-Cologne, Cologne, Germany; 5 Department of Infectious Diseases, Internal Medicine III, University Hospital of Ulm, Ulm, Germany; 6 Department of Internal Medicine, Goethe University Frankfurt, University Hospital Frankfurt, Infectious Diseases, Frankfurt, Germany; 7 Department of Medicine III, University Hospital, Ludwig-Maximilians- Universität (LMU) Munich, Munich, Germany; 8 Medical Faculty, Department of Haematology, Oncology and Cell Therapy, Otto von Guericke University Magdeburg, Magdeburg, Germany; 9 Department of Haematology, Oncology, and Clinical Immunology, Medical Faculty, University Hospital Düsseldorf, Heinrich-Heine-University Düsseldorf, Düsseldorf, Germany; 10 Center for Integrated Oncology Aachen Bonn Cologne Düsseldorf (CIO ABCD), Düsseldorf, Germany; 11 Faculty of Medicine, Institute for Digital Medicine and Clinical Data Science, Goethe University Frankfurt, Frankfurt, Germany; 12 Department I of Internal Medicine, Division of Infectious Diseases, Faculty of Medicine, University Hospital Cologne, University of Cologne, Kerpener Straße 62, 50937 Cologne, Germany

**Keywords:** Isavuconazole, Invasive mucormycosis, Invasive aspergillosis, Healthcare resource utilization, Real-world data, Cost analysis

## Abstract

**Purpose:**

Isavuconazole is effective against invasive aspergillosis (IA) and mucormycosis (IM) and may improve clinical outcomes compared to alternative antifungal treatments. However, real-world evidence regarding its clinical use and the health economic burden of inpatient treatment for IA and IM of patients with haematological malignancies remains limited.

**Methods:**

A retrospective, matched, multicentre cohort study was conducted in six German tertiary care centres. The study included adults with haematological or oncological diseases who were diagnosed with proven, probable, or possible IA or IM. We compared clinical and health economic outcomes under first-line treatment initiated with isavuconazole (case group) vs. liposomal amphotericin B (L-AmB) and/or voriconazole (control group) between 2016 and 2021. A micro-costing approach was used to assess direct treatment costs.

**Results:**

We included 198 patients (99 per group), most with a probable or possible classification. Median length of hospital stay was 44 days (interquartile range [IQR] 27–74) in the isavuconazole group and 39 days (IQR 26–56) in the control group (*p* = 0.285). All-cause mortality rates were 29% and 31% (*p* = 0.530), with fungal-related deaths occurring in 21% (*n* = 6) and 23% (*n* = 7, *p* = 0.862), respectively. Mean antifungal drug acquisition and overall treatment costs were significantly higher in the isavuconazole group (€22,389 vs. €12,801, *p* = 0.003; €49,042 vs. €39,369, *p* = 0.030, respectively), while mean hospitalisation costs were comparable (€28,570 vs. €31,160, *p* = 0.406).

**Conclusion:**

Our real-world analysis confirmed that first-line treatment initiated with isavuconazole resulted in clinical outcomes equivalent to those of L-AmB and/or voriconazole in patients with IA or IM. However, treatment costs during the in-patient stay were higher with isavuconazole.

**Supplementary Information:**

The online version contains supplementary material available at 10.1007/s15010-025-02674-x.

## Introduction

The treatment of invasive fungal diseases (IFDs), such as invasive aspergillosis (IA) and mucormycosis (IM), is challenging due to emerging resistance and the need for early therapeutic intervention [1–3]. IFDs are life-threatening infections that particularly affect immunocompromised individuals, with mortality rates reaching up to 75% [4–8]. A major reason for fatal outcomes is the considerable low rate of response to first-line treatment (e.g., due to nephrotoxicity), which often necessitates salvage therapy with a poor prognosis [9, 10]. The incidence of IM, which is also known for high morbidity and mortality, has increased over the past years [8, 11]. In addition to the clinical burden of IFDs, recent studies have demonstrated high treatment costs [12, 13]. Additionally, the rising prevalence of immunocompromised patients and the overall increase in IFDs underscore the urgent need to develop new treatment strategies [8].

Isavuconazole, a triazole antifungal agent, has a broad spectrum of activity against many pathogenic fungi and a favourable side-effect profile [2, 14–16]. It was approved in 2015 by the U.S. Food and Drug Administration (FDA) for treating IA and IM and by the European Medicines Agency (EMA) for IA and IM in patients for whom L-AmB is not appropriate [16–19]. The pivotal phase III SECURE trial demonstrated that isavuconazole is non-inferior to voriconazole for the primary treatment of IA and has a better safety profile. For IM, the single-arm phase III VITAL trial showed favourable efficacy for isavuconazole compared to a matched cohort of patients treated with L-AmB [17, 18].

Despite this, real-world data on the clinical and health economic outcomes of isavuconazole in treatment of IA and IM, particularly outside of clinical trials, remain scarce [8, 17]. To date, only a few studies have modelled its cost-effectiveness based on the SECURE and VITAL trials [13, 20–22]. We therefore performed a multicentre, retrospective matched cohort study to evaluate the clinical effectiveness and health economic burden of isavuconazole as a first-line treatment for IA and IM of patients with haematological malignancies in a real-world setting in Germany.

## Methods

### Study design and study population

This retrospective, multicentre cohort study was conducted at six German tertiary care centres with extensive experience in the treatment of haematological and oncological diseases. We applied modified intention-to-treat approach using a matched-pair design with a 1:1 ratio, comparing patients who received first-line treatment with isavuconazole (case group) to those treated with L-AmB or voriconazole (control group) between January 2016 and May 2021.

We included patients at the age of 18 years or older with a haematological or oncological disease and the evidence of proven, probable, or possible IA or IM, as defined by the 2008EORTC/MSG revised criteria [23]. Matching was performed based on the concordance of the following criteria in descending order of priority: (i) type and grade of fungal infection; (ii) APACHE (Acute Physiology and Chronic Health Evaluation) II score, if treated on intensive care unit (ICU); (iii) underlying condition favouring fungal infection (haematopoietic cell transplantation [HCT], aplasia following chemotherapy, other forms of immunosuppression, other reason); (iv) age; and (v) sex. The study was conducted in line with the Strengthening the Reporting of Observational Studies in Epidemiology (STROBE) statement, the Consolidated Health Economic Evaluation Reporting Standards 2022 (CHEERS), as well as national and international recommendations for health economic evaluation [24–27].

### Study objectives

The primary objective was to compare the clinical effectiveness and overall direct treatment costs of patients with IA or IM receiving first-line treatment with isavuconazole vs. L-AmB or voriconazole in a real-world setting. Clinical outcomes included risk factors for IFD, grade of IA and IM, clinical symptoms, diagnostic tests, and length of hospitalisation. The health economic analysis assessed the direct costs incurred during the hospital stay.

### Data collection and management

Data collection was performed retrospectively after regular hospital discharge or death by using data from standard-of-care medical records. Data were documented into a web-based electronic case report form (eCRF) in anonymised form accessible via www.clinicalsurveys.net, by internal medicine specialists at each participating centre. Data monitoring and query management were centrally performed by the lead study site, University Hospital Cologne.

### Healthcare resource utilization and cost analysis

The healthcare resource utilization and cost analysis was conducted from the perspective of the German healthcare system [26]. Costs for treatment on hospital ward were calculated based on the German Diagnosis Related Groups (G-DRG) systematic provided by Institute for the Hospital Renumeration System (InEK, Institut für das Entgeltsystem im Krankenhaus) and represented personnel and material costs for the years 2016 to 2021 [28]. Drug acquisition costs for IFD treatment were calculated by using the pharmacy retail price from 2024 extracted from the Rote Liste^®^, a comprehensive German drug directory [29]. To ensure comparability of hospitalisation and antifungal drug acquisition costs, the micro-costing approach used year 2024 values expressed in Euros (€). Indirect costs, such as productivity losses due to illness related disability or death before retirement age, were excluded from the analysis due to the severity of haematological and oncological diseases.

### Statistical analysis

RStudio version 4.5.0 (from 2025-04-11) was used for data analysis. Descriptive statistics were reported as mean with 95% confidence interval (95% CI) and median with interquartile range (IQR) and range. Sociodemographic and clinical differences between the case and control groups were assessed using the Pearson’s chi-squared test, Mann-Whitney-U test, log rank test or Student’s t-test (two-sided), as appropriate, applying a significance level of *p* < 0.05. Non-normally distributed cost values were analysed and compared by using Welch’s bootstrapped t-test with 10,000 replications and a starting point for the Mersenne twister at 1,000. A multivariate generalised linear model (GLM) with gamma distribution and log-link function was used to identify predictors of overall direct treatment costs. Results from the GLM were reported as odds ratios (OR) with 95% CIs. Only predictors with a significance level of *p* ≤ 0.1 in univariate regression analyses were evaluated in the GLM.

## Results

### Study population

A total of 198 patients with IA or IM were included in this retrospective matched cohort study. Ninety-nine cases treated with isavuconazole were matched with 99 controls that received L-AmB or voriconazole as first-line treatment. For seven patients in the case group, no suitable patients in the control group could be identified at the same study site and therefore they were matched with patients in the control group from other study sites. Patient characteristics are presented in Table 1, with the classification of IFD being the only variable showing significant differences between the two groups (*p* = 0.039).

**Table 1:** Patient characteristics of the total study population

	Isavuconazole group (*n* = 99)	Control group (*n* = 99)	*p* value
**Age in years; Median (Range)**	57 (19–86)	59 (28–77)	0.306^a^
**Female sex; n (%)**	34 (34)	32 (32)	0.763^b^
**Ethnic origin; n (%)**			1.000^c^
Caucasian	91 (92)	92 (93)	
Asian	2 (2)	2 (2)	
Sub-saharan African/Afro-American	1 (1)	0 (0)	
**Body Mass Index;** **Mean (95% CI)**	24.1 (23.2–25.1)	24.3 (23.4–25.2)	0.732^d^
Risk factor**; n (%)			
Neutropenia	46 (46)	37 (37)	0.195^b^
Immunosuppressive therapy	38 (38)	45 (45)	0.313^b^
Allogeneic HCT	32 (32)	23 (23)	0.153^b^
Acute kidney injury	23 (23)	19 (19)	0.487^b^
Intensive Care Treatment	15 (15)	13 (13)	0.683^b^
Diabetes mellitus	13 (13)	13 (13)	1.000^b^
Lymphocytopenia	12 (12)	14 (14)	0.674^b^
Chronic pulmonary disease	8 (8)	11 (11)	0.469^b^
Invasive ventilation	10 (10)	7 (7)	0.447^b^
Acute GvHD grade 1–2	13 (13)	4 (4)	0.224^b^
Acute GvHD grade 3–4	6 (6)	5 (5)	0.756^b^
Chronic graft versus host disease	7 (7)	4 (4)	0.352^b^
Autologous HCT	2 (2)	6 (6)	0.149^b^
**Underlying haematological/ oncological disease; n (%)**			0.711^b^
Haematological	95 (96)	95 (96)	
Oncological	3 (3)	4 (4)	
**Haematological disease; n (%)**	95 (96)	95 (96)	0.267^b^
Acute myeloid leukaemia	44 (46)	49 (52)	
Non-Hodgkin-Lymphoma	13 (14)	12 (13)	
Acute lymphoblastic leukaemia	8 (8)	5 (5)	
Chronic lymphoblastic leukaemia	7 (7)	8 (8)	
Multiple Myeloma	2 (2)	7 (7)	
Other	21 (22)	13 (14)	
**Invasive fungal disease; n (%)**			
Invasive aspergillosis	88 (89)	92 (93)	0.323^b^
Invasive mucormycosis	13 (13)	7 (7)	0.157^b^
**Classification of invasive mycosis; n (%)**			0.039^b^*
Possible	34 (34)	33 (33)	
Probable	44 (44)	57 (58)	
Proven	21 (21)	9 (9)	
Host factors***; n (%)			
Neutropenia (neutrophil cells < 500 µl)	60 (61)	62 (63)	0.840^b^
Immunosuppressive therapy	51 (52)	54 (55)	0.725^b^
Persistent fever (> 96 h)	28 (28)	36 (36)	0.243^b^
Therapy with corticosteroids ( > = 2 weeks)	24 (24)	15 (15)	0.100^b^
Signs and symptoms of GvHD after allogeneic HCT	18 (18)	13 (13)	0.313^b^
**Mycological criteria; n (%)**			
Conventional mycological examinations	76 (77)	75 (76)	0.766^b^
Non-culture-based antigen tests and molecular methods	70 (71)	78 (79)	0.232^b^
Tissue samples and liquid materials	85 (86)	91 (92)	0.238^b^

Abbreviations: CI, Confidence Interval; GvHD, graft-versus-host-disease; HCT, hematopoietic cell transplantation

^a^ Mann-Whitney U-test

^b^ Pearson chi-square test (two-tailed)

^c^ Fisher´s exact test

^d^ Student t-test

*p-value < 0.05

** Risk factors denote conditions or exposures present prior to the diagnosis of invasive aspergillosis or mucormycosis

*** Host factors are defined as conditions present at the time of diagnosis of invasive aspergillosis or mucormycosis

Across both groups, the most common risk factors for IA or IM were neutropenia, immunosuppressive therapy, allogeneic HCT and acute kidney injury. 96% of the patients in both groups had an underlying haematological disease, with acute myeloid leukaemia and non-Hodgkin lymphoma being the most prevalent diagnoses. Most of the patients in both groups had IA, while IM was diagnosed in 13% (*n* = 13) of patients in the case group compared to 7% (*n* = 7) in the control group with proven and probable cases only (*p* = 0.157). Two patients (2%) in the isavuconazole group had a concurrent diagnosis of both, IA and IM. The most commonly reported symptoms of IM and IA patients in the isavuconazole and control group were fever (64% vs. 68%), cough (30% vs. 30%), dyspnoea (29% vs. 30%), and pain (15% vs. 16%).

### Treatment and hospitalisation

The median length of hospital stay (LOS) was 44 days (IQR 27–74 days) in the isavuconazole group and 39 days (IQR 26–56 days) in the control group (*p* = 0.091, Table 2). Median treatment durations in the intermediate care unit and ICU did not differ between both groups, while patients in the control group required significantly longer durations of mechanical ventilation compared to the isavuconazole group.

**Table 2:** Hospitalisation and treatment details

	Isavuconazole group (*n* = 99)	Control group (*n* = 99)	*p* value
**Hospitalisation**^**I**^; **n (%)**	97 (98)	98 (99)	1.000^d^
Total days; Median (IQR)	44 (27–74)	39 (26–56)	0.091^a^
**Normal ward; n (%)**	89 (90)	84 (85)	0.285
Total days; Median (IQR)	38 (22–67)	33 (19–50)	0.161^a^
**Intermediate care unit; n (%)**	34 (34)	38 (38)	0.555
Total days; Median (IQR)	6 (3–12)	8 (2–27)	0.529^a^
**Intensive care unit; n (%)**	41 (41)	43 (43)	0.774
Total days; Median (IQR)	15 (6–22)	15 (6–29)	0.778^a^
**Mechanical ventilation; n (%)**	24 (24)	30 (30)	0.338
Total hours; Median (IQR)	233 (41–448)	358 (226–697)	0.044^a^*
**Antifungal treatment**			
**Isavuconazole**^**I**^; **n (%)**	92 (93)	-	-
Duration (days); Median (IQR)	12 (5–23)	-	-
**Liposomal Amphotericin B; n (%)**	47 (47)	60 (61)	0.064^b^
Duration (days); Median (IQR)	20 (10–33)	13 (8–28)	0.551^a^
**Voriconazole; n (%)**	31 (31)	67 (68)	< 0.001^b^***
Duration (days); Median (IQR)	9 (6–27)	12 (4–29)	0.111^a^

Abbreviations: IQR, interquartile range

^I^ *n*-values differ due to missing data

^a^ Mann-Whitney U-test

^b^ Pearson chi-square test (two-tailed)

^c^ Student t-test

^d^ Fisher´s exact test

* *p*-value < 0.05; *** *p*-value < 0.001

Patients in the case group were treated with isavuconazole orally (*n* = 52; 53%) or intravenously (*n* = 63; 64%) for an average of 12 days (IQR 5–23). For seven patients, no data were provided regarding the duration and dosage of isavuconazole and were therefore not considered in cost analyses. Notably, treatment regimens often evolved during hospitalisation. 47% of patients were treated with L-AmB and 31% received voriconazole after first-line treatment with isavuconazole during their hospital stay. While 31% (*n* = 31) of the patients were treated exclusively with isavuconazole, 17% received all of the three antifungal agents during the observation period. Patients in the control group received exclusively L-AmB and voriconazole of which 32% (*n* = 32) received both drugs. The durations of each antifungal therapy are shown in Table 2 for the whole study population, in Table S1 for the subgroup of patients with IA and in Table S2 for the subgroup of patients with IM.

### Clinical effectiveness

Antifungal treatment was stopped at discharge in 36% (*n* = 36) and 39% (*n* = 39) in the isavuconazole and control group (*p* = 0.451). The response to antifungal therapy was comparable between the two groups across all observation timepoints when comparing the total study population (Table 3). Data were most complete at day 14: 64% (*n* = 47) of patients in the isavuconazole group and 70% (*n* = 48) in the control group achieved at least stable clinical disease, whereas treatment failure occurred in 36% (*n* = 26) and 29% (*n* = 20) of patients, respectively (*p* = 0.098). Since therapeutic drug monitoring (TDM) for isavuconazole is not routinely performed in clinical practice, sufficient data on this aspect could not be retrieved. TDM for voriconazole was performed in 8% (*n* = 8) in the isavuconazole and 19% (*n* = 19) in the voriconazole group. Among those patients, TDM was performed a median of four times in the isavuconazole group and three times in the control group. The median voriconazole drug levels were 1,000 mg/L (IQR 227–2,408) in the isavuconazole group and 2,246 mg/L (IQR 1,000–5,853, *p* < 0.001) in the control group. Only adverse events attributed to the treatment with isavuconazole by the responsible investigator were recorded. Those were observed in three patients (3%, *n* = 3), all of whom had to discontinue the treatment with isavuconazole subsequently. Documented adverse events were kidney dysfunction in the first, confusion and disorientation in the second, and erythema and facial oedema in the third patient.

**Table 3:** Response to antifungal treatment and outcome

	Isavuconazole group (*n* = 99)	Control group (*n* = 99)	*p* value
**Therapy response on day 14; n (%)**	73 (74)	68 (69)	0.098^a^
Complete remission	6 (8)	9 (13)	
Partial remission	11 (15)	20 (29)	
Clinical stabilisation	30 (41)	19 (28)	
Therapy failure	26 (36)	20 (29)	
**Therapy response on day 28; n (%)**	49 (49)	43 (43)	0.976^a^
Complete remission	8 (16)	7 (16)	
Partial remission	15 (31)	13 (30)	
Clinical stabilisation	13 (27)	10 (23)	
Therapy failure	13 (27)	13 (30)	
**Therapy response on day 42; n (%)**	32 (32)	24 (24)	0.682^a^
Complete remission	8 (25)	4 (17)	
Partial remission	13 (41)	8 (33)	
Clinical stabilisation	6 (19)	6 (25)	
Therapy failure	5 (16)	6 (25)	
**Therapy response on day 84; n (%)**	15 (15)	13 (13)	0.317^b^
Complete remission	7 (47)	5 (39)	
Partial remission	8 (53)	5 (39)	
Clinical stabilisation	0 (0)	1 (8)	
Therapy failure	0 (0)	2 (15)	
**End of therapy response; n (%)**	26 (26)	34 (34)	0.563^b^
Complete remission	12 (46)	10 (29)	
Partial remission	1 (4)	3 (9)	
Clinical stabilisation	1 (4)	1 (3)	
Therapy failure	12 (46)	20 (59)	
**Antifungal treatment stopped at discharge; n (%)**	36 (36)	39 (39)	0.451^a^
**Outcome; n (%)**			
Regular discharge	63 (64)	58 (59)	0.411^a^
Transfer to other institution	6 (6)	10 (10)	0.307^a^
Death	29 (29)	31 (31)	0.793^a^

^a^ Pearson chi-square test (two-tailed)

^b^ Fisher´s exact test

In-hospital mortality was similar in both groups (29%, *n* = 29 vs. 31%, *n* = 31, *p* = 0.793). Among the deceased, IM or IA was considered the reason of death in 21% (*n* = 6) and 23% (*n* = 7, *p* = 0.862). In patients with IM, mortality was notably lower in the isavuconazole group (15%, *n* = 2) compared to the control group (57%, *n* = 4, *p* = 0.052). For IA patients, approximately one third died during their hospital stay in both groups (32%, *n* = 28 vs. 29%, *n* = 27, *p* = 0.681).

A comparison of treatment durations and outcomes between case and control patients with IA (Table S1 and S3) and those with IM (Table S2 and S4), respectively, revealed no significant differences.

### Hospitalisation and antifungal treatment costs

Direct cost factors, including hospitalisation and treatment costs, can be found in Table 4. After adjusting to 2024 values, the hospital stay was the most expensive factor in both groups. Overall antifungal drug acquisition costs were significantly higher in the isavuconazole group, with an average cost difference of €9,588 per patient, compared to the control group. Notably, drug acquisition costs for voriconazole were significantly lower in the isavuconazole compared to the control group (€2,035, 95% CI €1,030-€3,309 vs. €5,551, 95% CI €3,519-€7,980, *p* = 0.002) With €49,042 (95% CI €42,475-€56,449), the overall treatment costs were significantly higher in the isavuconazole group compared to the control group with €39,369 (95% CI €34,437-€44,826, *p* = 0.030). Among the 31 patients treated exclusively with isavuconazole, mean drug acquisition costs per patient were at €9,532 (95% CI €5,335-€14,979).

**Table 4:** Costs for hospitalisation and treatment in Euro

	Isavuconazole group (*n* = 99)	Control group (*n* = 99)	*p* value^a^
**Hospitalisation costs**^**I, b**^; **n (%)**	91 (92)	86 (87)	0.249^d^
Costs per patient; Mean (95% CI)	30,440 (25,210 − 35,670)	31,528 (26,110 − 36,945)	0.781
**Hospitalisation costs (Year 2024)**			
Costs per patient; Mean (95% CI)	28,570 (24,592 − 32,548)	31,160 (26,238 − 36,083)	0.406
**Antifungal drug acquisition costs** ^**c**^ Costs per patient; mean (95% CI)			
Isavuconazole	10,785 (8,364 − 13,518)	-	-
Liposomal Amphotericin B	9,689 (6,379 − 13,337)	7,250 (5,202-9,614)	0.253
Voriconazole	2,035 (1,030 − 3,309)	5,551 (3,519-7,980)	0.002**
**Overall antifungal drug acquisition costs**^**c**^; **n (%)**	90 (91)	82 (83)	0.092^d^
Costs per patient; Mean (95% CI)	22,389 (17,587 − 27,525)	12,801 (9,589 − 16,823)	0.003**
**Overall direct treatment costs**^**I, b, c**^; **n (%)**	91 (92)	86 (87)	0.249^d^
Costs per patient; Mean (95% CI)	50,912 (43,418 − 59,451)	39,736 (34,242 − 45,675)	0.026*
**Overall direct treatment costs (Year 2024)**			
Costs per patient; Mean (95% CI)	49,042 (42,475 − 56,449)	39,369 (34,437 − 44,826)	0.030*

Abbreviations: CI, Confidence Interval

^I^ *n*-values differ due to missing data

^a^ Bootstrapped t-test (independent samples, two sided)

^b^ Based on G-DRGs from 2016 to 2021

^c^ Based on pharmacy retail prices from Rote Liste^®^ 2024

^d^ Pearson chi-square test (two-tailed)

* *p*-value < 0.05; ** *p*-value < 0.01

To identify cost differences among different IFDs, detailed cost analyses for patients with IA (*n* = 180) and IM (*n* = 20) were provided in Table S5 and Table S6. Costs in IA patients were comparable to those observed in the total study population. Regarding the subgroup of patients with IM, hospitalisation costs remained similar, while drug acquisition costs and overall treatment costs were higher compared to the total study population. When regarding costs separated by classification of IFD, overall costs in the subgroup of patients with proven IFD were with €59,156 (95% CI 47,457 − 71,674) in the isavuconazole and €49,041 (95% CI 33,997 − 65,484) in the control group higher compared to the total study population and the subgroup of patients with IM (*p* = 0.332) due to both higher hospitalisation and antifungal drug acquisition costs.

The GLM presented predictors influencing overall direct treatment costs for patients with IA and IM (Table 5). Intensive care treatment, a longer hospital LOS, as well as treatment with isavuconazole or L-AmB were independently associated with significantly higher overall direct treatment costs.

**Table 5:** Generalised linear model of variables influencing overall direct treatment costs of total study population

Overall direct treatment costs	Univariate GLM			Multivariate GLM			
	OR	95% CI	*p*	OR	95% CI	*p*	
Age	0.992	0.985–0.999	0.033*	1.000	0.995–1.005	0.976	
Sex (Reference = Male)							
Female	0.909	0.735–1.131	0.387	-	-	-	
Body Mass Index	1.000	0.977–1.024	0.995	-	-	-	
IFD (Reference = IA)							
IM	1.302	0.937–1.869	0.135	-	-	-	
Classification of IFD (Reference = Possible IFD)							
Probable IFD	1.323	1.056–1.653	0.015*	1.142	0.972–1.341	0.106	
Proven IFD	1.579	1.168–2.159	0.004**	1.226	0.987–1.531	0.072	
Acute kidney injury	1.059	0.835–1.359	0.643				
Intensive care treatment	1.515	1.248–1.844	< 0.001***	1.466	1.267–1.698	< 0.001***	
Hospital LOS	1.012	1.009–1.014	< 0.001***	1.010	1.008–1.013	< 0.001***	
Isavuconazole treatment	1.316	1.085–1.596	0.006**	1.178	1.017–1.363	0.028*	
Liposomal Amphotericin B treatment	1.283	1.051–1.567	0.015*	1.243	1.074–1.438	0.004**	
Voriconazole treatment	1.166	0.956–1.421	0.131				
Response to antifungal treatment on day 14 (Reference = Complete remission)							
Partial remission	0.720	0.443–1.139	0.172	-	-	-	
Clinical stabilisation	1.042	0.657–1.594	0.856	-	-	-	
Therapy failure	1.102	0.694–1.693	0.669	-	-	-	

Note: *n* = 174; Akaike information criterium: 3,910; Bayes information criterium: 3,939; deviation: 40.7; likelihood quotient chi-square: 116.5 (*p* < 0.001)

Abbreviations: CI, confidence interval; GLM, generalised linear model; IA, invasive aspergillosis; IFD, invasive fungal disease; IM, invasive mucormycosis; OR, Odds ratio

* *p* value < 0.05; ** *p*-value < 0.01; *** *p*-value < 0.001

## Discussion

In this retrospective case-control study, we analysed the clinical effectiveness and health economic impact of first-line treatment with isavuconazole in patients with IA and IM treated in tertiary care centres in Germany. While clinical outcomes such as the hospital LOS, mortality, and response to antifungal treatment were comparable between the two groups, drug acquisition costs and overall direct treatment costs were significantly higher in the isavuconazole group compared to the control group. Notably, overall treatment of patients with IM was about €12,100 and €4,300 more expensive compared to treatment of patients with IA in the case and control groups, respectively. The most significant cost drivers were intensive care treatment, the hospital LOS and initiating treatment with L-AmB and isavuconazole.

Previous studies found similar clinical outcomes in patients with IFD treated with isavuconazole compared to those treated with voriconazole and/or L-AmB [17, 18, 30]. In the SECURE trial, mortality rates at day 84 of 28% and 29% are comparable to our study results [17]. Moreover, treatment success rates (partial and complete) at the end of therapy of 35% and 36% are comparable to our findings with 50% and 38%, with a higher success rate in our isavuconazole-treated population [17]. The VITAL trial, which compared treatment with isavuconazole to L-AmB in IM patients, found mortality rates at day 42 of 33% and 41% (*p* = 0.595) and complete and partial responses at the end of treatment of 14% and 17% in the total study population [18]. Based on patients enrolled in the SECURE trial, a prospective study from Horn et al. observed a shorter hospital LOS with a median of 13 and 15 days in the isavuconazole and voriconazole group compared to our study population (44 vs. 39 days) [31]. This is likely because our study population consisted exclusively of patients with haematological or oncological diseases requiring prolonged inpatient treatment for their underlying condition, whereas in the referenced study, only a proportion of patients had haematological malignancies. In the SECURE trial, the treatment durations in the intention-to-treat population were higher for isavuconazole and voriconazole, with a median of 45 (IQR 13–83) and 47 (IQR 13–83) days compared to our analysis with 12 (IQR 5–23) and 12 (IQR 4–29) days in the respective groups [17]. This may be due to treatment changes in cases of non-response. At day 14, 36% of patients in the isavuconazole group experienced therapy failure, resulting in a therapy switch to voriconazole or L-AmB. Less frequently, therapy changes were due to adverse events attributed to the treatment with isavuconazole in three patients. Additionally, the real-world design of our study may have contributed, as ongoing therapies could not always be continued following patient discharge. Nevertheless, we did not detect significant differences in therapy response between the two study groups.

These trials are characterised by a randomised-controlled study design, but real-world data on the clinical outcomes of IFD after treatment with isavuconazole remain limited. To date, few observational studies have been published that reported similar [32] or even better [33] clinical outcomes when treating IFDs with isavuconazole compared to other antifungal drugs. Other studies were only descriptive, focusing on clinical experience and adverse event rates of isavuconazole without comparison to other antifungal drugs [3, 34–38]. Our study contributes new insights into the clinical effectiveness of isavuconazole compared to voriconazole or L-AmB in treating IFDs in a real-world, multicentre design.

In our study, hospitalisation costs for patients initiating first-line treatment with isavuconazole were comparable to those of patients treated with L-AmB and/or voriconazole. The economic model from Azanza et al. also indicated that hospitalisation costs were comparable in the groups, while drug acquisition costs and total costs were about €5,000 higher in the isavuconazole-treated group [21]. Their reported drug acquisition cost for isavuconazole (€12,883 per patient) was similar to our findings, while their cost for voriconazole (€7,644) was higher [21]. The economic analysis from Moya-Alarcón et al. described cost savings per patient treated with isavuconazole of €5,038 compared to treatment with liposomal L-AmB, which was attributed to a shorter duration in intravenous treatment [2]. Bagshaw et al. came to similar conclusions, reporting lower treatment costs of £3,770 or 20% in their groups (isavuconazole group: £14,842 per patient vs. L-AmB group: £18,612 per patient) [30]. When interpreting the results, it is important to consider that our study also included patients treated with voriconazole in the control group, which has substantially lower drug acquisition costs than isavuconazole and L-AmB. Besides L-AmB, which was plausibly identified as a cost driver in our GLM due to its high acquisition costs, our study confirms the high financial burden of isavuconazole treatment compared to generic voriconazole. This finding aligns with results from other studies and is largely driven by the drug’s ongoing patent protection [21].

Nevertheless, several studies have suggested that isavuconazole is cost-effective compared to voriconazole at a willingness-to-pay threshold (WPT) of €25,000/quality-adjusted life year (QALY) in Spain [21], $50,000 per death avoided in the United States [13] and £30,000/QALY in the United Kingdom [22]. The cost-effectiveness analysis from Azanza et al. conducted in Spain demonstrated that treatment with isavuconazole leads to 0.49 more life year gains (LY) and 0.41 more QALYs compared to treatment with voriconazole in patients with IA and IM [21]. Another study conducted in the United Kingdom came to similar conclusions (LYs = 0.48; QALYs = 0.39). In the deterministic sensitivity analyses from Harrington et al., isavuconazole was associated with lower total costs per treated patient than voriconazole, except for when the initial hospital LOS was increased by 25% in the isavuconazole group [13]. These results emphasise our findings in identifying hospital LOS as a key cost driver. Additionally, when focusing exclusively on patients treated with isavuconazole monotherapy in our study, mean antifungal drug acquisition costs per patient of €9,532 were lower compared to those who also received voriconazole and/or liposomal amphotericin B (€22,389). Consequently, overall antifungal drug acquisition costs in this subgroup were lower than those observed in the control group (€12,801).

Our findings demonstrated higher drug acquisition and overall treatment costs for IM patients compared to IA patients, suggesting that IM is more challenging to treat due to the difficulty in pathogen detection, which is compounded by non-specific symptoms and the rapid progression of disease [22]. Definite diagnosis of proven and probable cases requires direct sampling of infected tissue [23]. Consequently, the need for multiple diagnostic measures and the initiation of treatment before definitive diagnosis contribute to higher treatment costs [12]. Since the proportion of patients with proven IFD was higher in the isavuconazole group, costs were also expected to be higher. Heimann et al. compared direct treatment costs from patients with proven or probable IM to matched patients with similar underlying conditions based on G-DRGs in a cost-of-illness analysis [12]. They concluded that mean costs of patients with IM were significantly higher compared to those of matched patients (€53,261, 95% CI 39,660 − 68,825 vs. €20,269, 95% CI 14,707 − 26,653, *p* < 0.001). Antifungal drug acquisition costs for IM patients in their study were €22,819 (95% CI 15,036 − 32,346), which is slightly higher than the costs in our control group (€18,127, 95% CI 19,103 − 26,101). When interpreting these numbers, it should be noted that the study from Heimann et al. also considered treatment costs with posaconazole and caspofungin [12].

Our analysis is subject to important limitations. First, and most critically, the retrospective matching did not result in fully comparable cohorts. The isavuconazole group contained a significantly higher proportion of patients with ‘proven’ IFD (21% vs. 9%), introducing a strong potential for selection bias that likely confounds both clinical and cost comparisons. Second, our analysis compares treatment strategies, not just single drugs. The high rate of treatment switching means the costs attributed to the ‘isavuconazole group’ also include expensive subsequent therapies, making direct cost comparisons challenging. Third, our economic analysis was limited to the index hospitalisation and could not capture potential downstream cost savings from oral step-down therapy, a key feature of isavuconazole. Fourth, the inclusion of a large number of ‘possible’ IFD cases, who may not have had a fungal infection, likely diluted any true differences in treatment efficacy. Fifth, as this is a real-world study, data regarding TDM of voriconazole and isavuconazole were not comprehensively collected. It is reasonable to assume that TDM was performed more frequently in clinical practice. As TDM was not the primary focus of this study, this limitation has not influenced the main study outcomes. Finally, our findings are from the German inpatient setting and may not be generalizable to other health care systems.

In conclusion, our study found that a treatment strategy starting with isavuconazole was associated with higher overall treatment costs for IA and IM patients compared to treatment with L-AmB and/or voriconazole, while clinical outcomes were comparable. To our knowledge, this is the first study comparing treatment costs for IM patients to those of IA patients in a real-world inpatient setting in Germany. Clinical practice should focus on improving treatment strategies and early detection of IFD [18], especially in IM patients, to (i) decrease infection incidence, the hospital LOS, and mortality, and to (ii) reduce the identified cost drivers.

## Supplementary Information

Below is the link to the electronic supplementary material.

Supplementary Material 1

Supplementary Material 2

## Data Availability

The authors are not permitted to disclose the study data beyond the research team. However, the data can be accessed through direct request to the corresponding author, and R scripts used to analyse the data can be made available on reasonable request.
