# Supplementary material for: Clinical and health economic impact of isavuconazole for treatment of invasive aspergillosis and mucormycosis: a retrospective, matched multicentre cohort study in Germany
Source: Infection. 2025 Oct 31;54(1):377–87. doi: 10.1007/s15010-025-02674-x (PMC12864223; doi:10.1007/s15010-025-02674-x)
Supplement: Supplementary file 1 — Supplementary Material 1 [file 15010_2025_2674_MOESM1_ESM.docx]

**Supplementary Material** - Clinical and health economic impact of isavuconazole for treatment of invasive aspergillosis and mucormycosis: A retrospective matched multicentre cohort study in Germany

**Table S1** Costs for hospitalisation and treatment in Euro in IA patients (n=180)

|  | **Isavuconazole group (n=88)** | **Control group (n=92)** | **p value^a^** |
| --- | --- | --- | --- |
| **Hospitalisation costs^I, b^; n (%)** | 80 (91) | 80 (87) | 0.399^d^ |
| Costs per patient;  Mean (95% CI) | 30,280 (24,644-35,916) | 31,300 (25,609-36,991) | 0.807 |
| **Hospitalisation costs (Year 2024)** | |  |  |
| Costs per patient;  Mean (95% CI) | 28,932 (24,608-33,255) | 31,293 (26,073-36,512) | 0.494 |
| **Antifungal drug acquisition costs^c^**  Costs per patient; Mean (95% CI) |  |  |  |
| Isavuconazole | 10,713 (8,120-13,630) | - | - |
| Liposomal Amphotericin B | 7,332 (4,468-10,923) | 6,417 (4,438-8,809) | 0.644 |
| Voriconazole | 2,171 (1,046-3,580) | 5,963 (3,860-8,542) | 0.003** |
| **Overall antifungal drug acquisition costs^c^; n (%)** | 80 (91) | 76 (83) | 0.101^d^ |
| Costs per patient; Mean (95% CI) | 20,081 (15,369-25,347) | 12,380 (9,051-16,649) | 0.016* |
| **Overall direct treatment costs^I, b, c^; n (%)** | 80 (91) | 80 (87) | 0.399^d^ |
| Costs per patient; Mean (95% CI) | 48,461 (40,519-57,553) | 39,077 (33,213-45,323) | 0.076 |
| **Overall direct treatment costs (Year 2024)** | |  |  |
| Costs per patient; Mean (95% CI) | 47,112 (40,171-55,135) | 39,070 (33,686-44,854) | 0.084 |
| Abbreviations: CI, Confidence Interval | | | |
| ^I^ n-values differ due to missing data | | | |
| ^a^ Bootstrapped t-test (independent samples, two sided) | | | |
| ^b^ Based on G-DRGs from 2016 to 2021 | | | |
| ^c^ Based on pharmacy retail prices from Rote Liste® 2024 | | | |
| ^d^ Pearson chi-square test (two-tailed) | | | |
| * p-value < 0.05; ** p-value < 0.01 | | | |

**Table S2** Costs for hospitalisation and treatment in Euro in IM patients (n=20)

|  | **Isavuconazole group (n=13)** | **Control group (n=7)** | **p value^a^** |
| --- | --- | --- | --- |
| **Hospitalisation costs^I, b^; n (%)** | 13 (100) | 6 (86) | 0.162^d^ |
| Costs per patient; Mean (95% CI) | 30,046 (16,191-43,901) | 34,560 (10,859-58,262) | 0.699 |
| **Hospitalisation costs (Year 2024)** | |  |  |
| Costs per patient; Mean (95% CI) | 25,864 (16,341-35,386) | 29,395 (12,274-46,516) | 0.655 |
| **Antifungal drug acquisition costs^c^**  Costs per patient; Mean (95% CI) |  |  |  |
| Isavuconazole | 10,982 (6,736-15,933) | - | - |
| Liposomal Amphotericin B | 24,349 (12,577-37,209) | 17,797 (9,208-25,518) | 0.395 |
| Voriconazole | 790 (0-1,977) | 330 (0-753) | 0.432 |
| **Overall antifungal drug acquisition costs^c^; n (%)** | 12 (92) | 6 (86) | 0.639^d^ |
| Overall costs per patient; Mean (95% CI) | 36,121 (23,314-49,704) | 18,127 (9,103-26,101) | 0.018* |
| **Overall direct treatment costs^I, b, c^; n (%)** | 13 (100) | 6 (86) | 0.162^d^ |
| Overall costs per patient; Mean (95% CI) | 63,388 (44,684-84,572) | 48,514 (33,296-60,757) | 0.226 |
| **Overall direct treatment costs (Year 2024)** | |  |  |
| Overall costs per patient; Mean (95% CI) | 59,206 (43,345-76,418) | 43,348 (30,527-53,391) | 0.120 |
| Abbreviations: CI, Confidence Interval | | | |
| ^I^ n-values differ due to missing data | | | |
| ^a^ Bootstrapped t-test (independent samples, two sided) | | | |
| ^b^ Based on G-DRGs from 2016 to 2021 | | | |
| ^c^ Based on pharmacy retail prices from Rote Liste® 2024 | | | |
| ^d^ Pearson chi-square test (two-tailed) | | | |
| * p-value < 0.05 | | | |
