# Supplementary material for: Clinical and health economic impact of isavuconazole for treatment of invasive aspergillosis and mucormycosis: a retrospective, matched multicentre cohort study in Germany
Source: Infection. 2025 Oct 31;54(1):377–87. doi: 10.1007/s15010-025-02674-x (PMC12864223; doi:10.1007/s15010-025-02674-x)
Supplement: Supplementary file 2 — Supplementary Material 2 [file 15010_2025_2674_MOESM2_ESM.docx]

**Supplementary Material** - Clinical and health economic impact of isavuconazole for treatment of invasive aspergillosis and mucormycosis: A retrospective matched multicentre cohort study in Germany

**Table S1** Hospitalisation and treatment details in patients with invasive aspergillosis (n = 180)

|  | **Isavuconazole group (n=88)** | **Control group (n=92)** | **p value** |
| --- | --- | --- | --- |
| **Hospitalisation^I^; n (%)** | 86 (98) | 91 (99) | 0.615^a^ |
| Total days; Median (IQR) | 44 (27-66) | 39 (28-56) | 0.204^b^ |
| **Normal ward; n (%)** | 78 (89) | 78 (85) | 0.447^c^ |
| Total days; Median (IQR) | 37 (21-59) | 33 (19-48) | 0.355^b^ |
| **Intermediate care unit; n (%)** | 28 (32) | 36 (39) | 0.306^c^ |
| Total days; Median (IQR) | 6 (3-14) | 9 (3-29) | 0.471^b^ |
| **Intensive care unit; n (%)** | 37 (42) | 40 (44) | 0.846^c^ |
| Total days; Median (IQR) | 15 (6-24) | 15 (6-30) | 0.907^b^ |
| **Mechanical ventilation; n (%)** | 21 (24) | 28 (30) | 0.322^c^ |
| Total hours; Median (IQR) | 291 (120-451) | 377 (209-710) | 0.149^b^ |
| **Antifungal treatment** |  |  |  |
| **Isavuconazole^I^; n (%)** | 82 (93) | - | - |
| Duration (days); Median (IQR) | 11 (5-22) | - | - |
| **Liposomal Amphotericin B; n (%)** | 37 (42) | 53 (58) | 0.037^c^* |
| Duration (days); Median (IQR) | 18 (10-30) | 13 (8-28) | 0.751^b^ |
| **Voriconazole; n (%)** | 28 (32) | 65 (71) | <0.001^c^*** |
| Duration (days); Median (IQR) | 11 (6-29) | 12 (4-29) | 0.097^b^ |
| Abbreviations: IQR, interquartile range | | | |
| ^I^ n-values differ due to missing data | | | |
| ^a^ Fisher´s exact test | | | |
| ^b^ Mann-Whitney U-test | | | |
| ^c^ Pearson chi-square test (two-tailed) | | | |
| * p-value <0.05; *** p-value <0.001 | | | |

**Table S2** Hospitalisation and treatment details in patients with invasive mucormycosis (n = 20)

|  | **Isavuconazole group (n=13)** | **Control group (n=7)** | **p value** |
| --- | --- | --- | --- |
| **Hospitalisation^I^; n (%)** | 13 (100) | 7 (100) | - |
| Total days; Median (IQR) | 78 (26-85) | 32 (18-57) | 0.205^a^ |
| **Normal ward; n (%)** | 13 (100) | 6 (86) | 0.162^b^ |
| Total days; Median (IQR) | 72 (26-79) | 40 (15-62) | 0.219^a^ |
| **Intermediate care unit; n (%)** | 7 (54) | 2 (29) | 0.374^c^ |
| Total days; Median (IQR) | 3 (3-7) | 3 (2-4) | 0.555^a^ |
| **Intensive care unit; n (%)** | 6 (46) | 3 (43) | 1.000^c^ |
| Total days; Median (IQR) | 7 (3-10) | 13 (8-17) | 0.437^a^ |
| **Mechanical ventilation; n (%)** | 4 (31) | 2 (29) | 1.000^c^ |
| Total hours; Median (IQR) | 22 (14-68) | 289 (283-294) | 0.105^a^ |
| **Antifungal treatment** |  |  |  |
| **Isavuconazole^I^; n (%)** | 12 (92) | - | - |
| Duration (days); Median (IQR) | 25 (6-29) | - | - |
| **Liposomal Amphotericin B; n (%)** | 12 (92) | 7 (100) | 0.452^b^ |
| Duration (days); Median (IQR) | 21 (8-26) | 15 (11-31) | 0.719^a^ |
| **Voriconazole; n (%)** | 3 (23) | 2 (29) | 1.000^c^ |
| Duration (days); Median (IQR) | 7 (5-15) | 13 (8-17) | 0.331^a^ |
| Abbreviations: IQR, interquartile range | | | |
| ^I^ n-values differ due to missing data | | | |
| ^a^ Mann-Whitney U-test | | | |
| ^b^ Pearson chi-square test (two-tailed) | | | |
| ^c^ Fisher´s exact test | | | |

**Table S3** Response to antifungal treatment and outcome in patients with invasive aspergillosis (n = 180)

|  | **Isavuconazole group (n=88)** | **Control group (n=92)** | **p value** |
| --- | --- | --- | --- |
| **Therapy response on day 14; n (%)** | 63 (72) | 64 (70) | 0.057^a^ |
| Complete remission | 6 (8) | 8 (13) |  |
| Partial remission | 8 (11) | 20 (31) |  |
| Clinical stabilisation | 25 (35) | 17 (27) |  |
| Therapy failure | 24 (33) | 19 (30) |  |
| **Therapy response on day 28; n (%)** | 41 (47) | 41 (45) | 0.977^a^ |
| Complete remission | 8 (20) | 7 (17) |  |
| Partial remission | 11 (27) | 12 (29) |  |
| Clinical stabilisation | 10 (24) | 9 (22) |  |
| Therapy failure | 12 (29) | 13 (32) |  |
| **Therapy response on day 42; n (%)** | 24 (27) | 22 (24) | 0.479^b^ |
| Complete remission | 8 (33) | 4 (18) |  |
| Partial remission | 8 (33) | 6 (27) |  |
| Clinical stabilisation | 3 (13) | 6 (27) |  |
| Therapy failure | 5 (21) | 6 (27) |  |
| **Therapy response on day 84; n (%)** | 12 (14) | 12 (13) | 0.496^b^ |
| Complete remission | 7 (58) | 5 (42) |  |
| Partial remission | 5 (42) | 4 (33) |  |
| Clinical stabilisation | 0 (0) | 1 (8) |  |
| Therapy failure | 0 (0) | 2 (17) |  |
| **End of therapy response; n (%)** | 24 (27) | 30 (33) | 0.815^b^ |
| Complete remission | 11 (46) | 10 (33) |  |
| Partial remission | 1 (4) | 2 (7) |  |
| Clinical stabilisation | 0 (0) | 1 (3) |  |
| Therapy failure | 12 (50) | 17 (57) |  |
| **Antifungal treatment stopped at discharge; n (%)** | 33 (38) | 34 (37) | 0.825^a^ |
| **Outcome; n (%)** |  |  |  |
| Regular discharge | 54 (61) | 55 (60) | 0.754^a^ |
| Transfer to other institution | 5 (6) | 10 (11) | 0.216^a^ |
| Death | 28 (32) | 27 (29) | 0.681^a^ |
| ^a^ Pearson chi-square test (two-tailed) | | | |
| ^b^ Fisher´s exact test | | | |

**Table S4** Response to antifungal treatment and Outcome in patients with invasive mucormycosis (n = 20)

|  | **Isavuconazole group (n=13)** | **Control group (n=7)** | **p value** |
| --- | --- | --- | --- |
| **Therapy response on day 14; n (%)** | 11 (85) | 4 (57) | 0.862^a^ |
| Complete remission | 1 (9) | 1 (25) |  |
| Partial remission | 3 (27) | 0 (0) |  |
| Clinical stabilisation | 5 (45) | 2 (50) |  |
| Therapy failure | 2 (18) | 1 (25) |  |
| **Therapy response on day 28; n (%)** | 8 (62) | 2 (29) | 1.000^a^ |
| Complete remission | 0 (0) | 0 (0) |  |
| Partial remission | 4 (50) | 1 (50) |  |
| Clinical stabilisation | 3 (38) | 1 (50) |  |
| Therapy failure | 1 (13) | 0 (0) |  |
| **Therapy response on day 42; n (%)** | 8 (62) | 2 (29) | 1.000^a^ |
| Complete remission | 0 (0) | 0 (0) |  |
| Partial remission | 5 (63) | 2 (100) |  |
| Clinical stabilisation | 3 (38) | 0 (0) |  |
| Therapy failure | 0 (0) | 0 (0) |  |
| **Therapy response on day 84; n (%)** | 3 (23) | 1 (14) | 1.000^a^ |
| Complete remission | 0 (0) | 0 (0) |  |
| Partial remission | 3 (100) | 1 (100) |  |
| Clinical stabilisation | 0 (0) | 0 (0) |  |
| Therapy failure | 0 (0) | 0 (0) |  |
| **End of therapy response; n (%)** | 2 (15) | 4 (57) | 0.200^a^ |
| Complete remission | 1 (50) | 0 (0) |  |
| Partial remission | 0 (0) | 1 (25) |  |
| Clinical stabilisation | 1 (50) | 0 (0) |  |
| Therapy failure | 0 (0) | 3 (75) |  |
| **Antifungal treatment stopped at discharge; n (%)** | 4 (31) | 5 (71) | 0.160^a^ |
| **Outcome; n (%)** |  |  |  |
| Regular discharge | 10 (77) | 3 (43) | 0.174^a^ |
| Transfer to other institution | 1 (8) | 0 (0) | 1.000^a^ |
| Death | 2 (15) | 4 (57) | 0.122^a^ |
| ^a^ Fisher´s exact test | | | |

**Table S5** Costs for hospitalisation and treatment in Euro in patients with invasive aspergillosis (n=180)

|  | **Isavuconazole group (n=88)** | **Control group (n=92)** | **p value^a^** |
| --- | --- | --- | --- |
| **Hospitalisation costs^I, b^; n (%)** | 80 (91) | 80 (87) | 0.399^d^ |
| Costs per patient;  Mean (95% CI) | 30,280 (24,644-35,916) | 31,300 (25,609-36,991) | 0.807 |
| **Hospitalisation costs (Year 2024)** | |  |  |
| Costs per patient;  Mean (95% CI) | 28,932 (24,608-33,255) | 31,293 (26,073-36,512) | 0.494 |
| **Antifungal drug acquisition costs^c^**  Costs per patient; Mean (95% CI) |  |  |  |
| Isavuconazole | 10,713 (8,120-13,630) | - | - |
| Liposomal Amphotericin B | 7,332 (4,468-10,923) | 6,417 (4,438-8,809) | 0.644 |
| Voriconazole | 2,171 (1,046-3,580) | 5,963 (3,860-8,542) | 0.003** |
| **Overall antifungal drug acquisition costs^c^; n (%)** | 80 (91) | 76 (83) | 0.101^d^ |
| Costs per patient; Mean (95% CI) | 20,081 (15,369-25,347) | 12,380 (9,051-16,649) | 0.016* |
| **Overall direct treatment costs^I, b, c^; n (%)** | 80 (91) | 80 (87) | 0.399^d^ |
| Costs per patient; Mean (95% CI) | 48,461 (40,519-57,553) | 39,077 (33,213-45,323) | 0.076 |
| **Overall direct treatment costs (Year 2024)** | |  |  |
| Costs per patient; Mean (95% CI) | 47,112 (40,171-55,135) | 39,070 (33,686-44,854) | 0.084 |
| Abbreviations: CI, Confidence Interval | | | |
| ^I^ n-values differ due to missing data | | | |
| ^a^ Bootstrapped t-test (independent samples, two sided) | | | |
| ^b^ Based on G-DRGs from 2016 to 2021 | | | |
| ^c^ Based on pharmacy retail prices from Rote Liste® 2024 | | | |
| ^d^ Pearson chi-square test (two-tailed) | | | |
| * p-value < 0.05; ** p-value < 0.01 | | | |

**Table S6** Costs for hospitalisation and treatment in Euro in patients with invasive mucormycosis (n=20)

|  | **Isavuconazole group (n=13)** | **Control group (n=7)** | **p value^a^** |
| --- | --- | --- | --- |
| **Hospitalisation costs^I, b^; n (%)** | 13 (100) | 6 (86) | 0.162^d^ |
| Costs per patient; Mean (95% CI) | 30,046 (16,191-43,901) | 34,560 (10,859-58,262) | 0.699 |
| **Hospitalisation costs (Year 2024)** | |  |  |
| Costs per patient; Mean (95% CI) | 25,864 (16,341-35,386) | 29,395 (12,274-46,516) | 0.655 |
| **Antifungal drug acquisition costs^c^**  Costs per patient; Mean (95% CI) |  |  |  |
| Isavuconazole | 10,982 (6,736-15,933) | - | - |
| Liposomal Amphotericin B | 24,349 (12,577-37,209) | 17,797 (9,208-25,518) | 0.395 |
| Voriconazole | 790 (0-1,977) | 330 (0-753) | 0.432 |
| **Overall antifungal drug acquisition costs^c^; n (%)** | 12 (92) | 6 (86) | 0.639^d^ |
| Overall costs per patient; Mean (95% CI) | 36,121 (23,314-49,704) | 18,127 (9,103-26,101) | 0.018* |
| **Overall direct treatment costs^I, b, c^; n (%)** | 13 (100) | 6 (86) | 0.162^d^ |
| Overall costs per patient; Mean (95% CI) | 63,388 (44,684-84,572) | 48,514 (33,296-60,757) | 0.226 |
| **Overall direct treatment costs (Year 2024)** | |  |  |
| Overall costs per patient; Mean (95% CI) | 59,206 (43,345-76,418) | 43,348 (30,527-53,391) | 0.120 |
| Abbreviations: CI, Confidence Interval | | | |
| ^I^ n-values differ due to missing data | | | |
| ^a^ Bootstrapped t-test (independent samples, two sided) | | | |
| ^b^ Based on G-DRGs from 2016 to 2021 | | | |
| ^c^ Based on pharmacy retail prices from Rote Liste® 2024 | | | |
| ^d^ Pearson chi-square test (two-tailed) | | | |
| * p-value < 0.05 | | | |
